# Draft Genome Sequence of Cryophilic Basidiomycetous Yeast *Mrakia blollopis* SK-4, Isolated from an Algal Mat of Naga-ike Lake in the Skarvsnes Ice-Free Area, East Antarctica

**DOI:** 10.1128/genomeA.01454-14

**Published:** 2015-01-22

**Authors:** Masaharu Tsuji, Sakae Kudoh, Tamotsu Hoshino

**Affiliations:** a Biomass Refinery Research Center (BRRC), National Institute of Advanced Industrial Science and Technology (AIST), Kagamiyama, Higashihiroshima, Hirosima, Japan; b National Institute of Polar Research (NIPR), Tachikawa, Tokyo, Japan

## Abstract

*Mrakia blollopis* strain SK-4 was isolated from an algal mat of Naga-ike, a lake in Skarvsnes, East Antarctica. Here, we report the draft genome sequence of M. blollopis SK-4. This is the first report on the genome sequence of any cold-adapted fungal species.

## GENOME ANNOUNCEMENT

The cryophilic yeasts (1) *Mrakia* spp. and *Mrakiella* spp. have been found in the Arctic, Alaska, the Italian Alps, Patagonia, and Antarctica (2). di Menna (3) reported that the genus *Mrakia* accounts for about 24% of the culturable yeast in Antarctic soil. Moreover, we previously reported that about 35% of culturable fungi isolated from lake sediment and soil of East Antarctica were *Mrakia* spp. (4). These reports suggested that *Mrakia* spp. are the dominant culturable fungi in East Antarctica and the most adaptive to the Antarctic area. *Mrakia blollopis* SK-4 was isolated from Naga-ike Lake in the Skarvsnes ice-free area, East Antarctica. This yeast strain was shown to have the ability to decompose milk fat and to ferment typical sugars, such as glucose, sucrose, maltose, raffinose, and fructose, at cold temperatures (5). Strain SK-4 can convert 120 g/liter glucose to 48.7 g/liter ethanol at 10°C, while other *Mrakia* spp. could not produce >2% (vol/vol) ethanol (2, 6). Moreover, strain SK-4 can completely convert glucose to ethanol at pH 4.0 to 10.0 at 10°C (7).

To the best of our knowledge, a genome sequence has not been published for any cold-adapted fungal species. Here, we report the draft genome sequence of M. blollopis SK-4.

M. blollopis SK-4 was inoculated into 400 ml of YPD liquid medium (10 g/liter yeast extract, 20 g/liter peptone, and 20 g/liter glucose) and cultured at 120 rpm for 120 h at 10°C. Next, 400 ml of the culture was collected by centrifugation at 3,500 × *g* for 10 min at 4°C. The SK-4 genomic DNA was extracted by using an ISOPLANT II kit (Wako Pure Chemical Industries) with Westase (TaKaRa Bio). Next, extracted genomic DNA was purified by NucleoBond AXG20/100 and Nucleo buffer set III (TaKaRa Bio), according to the manufacturer’s protocol. The concentration and purity of the genomic DNA were determined by NanoDrop (Thermo Scientific) and the Quant-iT double-stranded DNA (dsDNA) broad-range (BR) assay kit (Invitrogen). Next, 2.6 µg of the genomic DNA was digested by g-TUBE (Covaris), and the terminal of the digested DNA was smoothed by connecting a SMARTbell adaptor. A SMARTbell library was constructed using the DNA template prep kit 2.0 (3.0 to 10.0 kb) (Pacific Biosciences). The quality of the library was confirmed by the Quant-iT dsDNA BR assay kit, and the library size was checked using the Agilent 2200 TapeStation (Agilent Technologies, Inc.). A sequence template was prepared using the DNA/polymerase binding kit P-40 (Pacific Biosciences). The sequence reaction was performed on a PacBio RS II (140-fold coverage) (Pacific Biosciences), and assembly was conducted using SMRT Analysis version 2.1.1 (Pacific Biosciences) (8).

The genome size of M. blollopis SK-4 is 30,464,263 bases, and the G+C content is 53.71%. The final assembly consists of 167 contigs (*N*_50_, 1,718,111 bp; maximum length, 2,325,364 bp). The results of genome analysis showed that SK-4 genotype is haploid. We also predicted proteins and open reading frames (ORFs) using MAKER2 version 2.10 (9), Augustus version 3.0 (10), and NCBI BLAST version 2.2.20 (11). Consequently, we found 8,907 putative proteins. This is the first report on the genome sequence of any cold-adapted fungal species.

### Nucleotide sequence accession numbers.

The genome sequence of M. blollopis strain SK-4 has been deposited at DDBJ/EMBL/GenBank under the accession numbers BBPU01000001 to BBPU01000167. The version described in this paper is the first version.
